# Mechanical and Hemocompatibility Assessment of Selected Technologies for Prosthesis Connection with the Outflow Cannula Graft of the RH PED^®^ Pediatric Pulsatile Pump for Heart Support

**DOI:** 10.3390/jcm14134621

**Published:** 2025-06-30

**Authors:** Klaudia Cholewa, Przemysław Kurtyka, Karolina Janiczak, Artur Kapis, Agnieszka Szuber-Dynia, Mateusz Janecki, Witold Walke, Karolina Wilk, Maciej Gawlikowski

**Affiliations:** 1 Faculty of Biomedical Engineering, Silesian University of Technology, Roosevelta 40 Str., 41-800 Zabrze, Poland; matejan437@student.polsl.pl (M.J.); witold.walke@polsl.pl (W.W.); maciej.gawlikowski@polsl.pl (M.G.); 2 Institute of Heart Prostheses, Foundation of Cardiac Surgery Development, Wolności 345a Str., 41-800 Zabrze, Poland; pkurtyka@frk.pl (P.K.); janiczakkrj@gmail.com (K.J.); akapis@frk.pl (A.K.); aszuber@frk.pl (A.S.-D.)

**Keywords:** cardiac surgery, heart failure, mechanical circulatory support, pulsatile pumps, extracorporeal circulatory support pump, biocompatibility, hemocompatibility, biological tests, cannulas

## Abstract

**Background:** Heart failure in pediatric patients remains a major cause of morbidity and mortality, often associated with congenital heart defects and cardiomyopathies. Mechanical circulatory support (MCS) devices have emerged as critical therapeutic options, particularly as bridges to transplantation or recovery. The complexity of their use in children necessitates highly specialized solutions. This study aimed to evaluate the quality and performance of selected connection technologies between prosthetic vascular grafts and the outflow cannula of the Religa Heart PED^®^ pediatric pulsatile pump, with a focus on tightness, surface smoothness, and structural integrity. **Methods:** Mechanical testing was conducted on various connection types, including static tensile strength and long-term durability under pulsatile flow conditions with biological fluid analogs. Macro and microscopic evaluations assessed the surface quality and potential thrombogenic risks, biological testing encompassed permeability analysis in static and dynamic settings, and hemocompatibility was determined by acute thrombogenicity. Additionally, in vivo observations in a large animal model were used for final qualitative validation. **Results:** All connection types demonstrated sufficient mechanical strength, with no structural degradation or leakage observed in any samples following long-term testing. Thrombus formation was absent in adhesive connections with Dacron and polytetrafluoroethylene (PTFE) grafts but was observed in the mechanical connection with the PTFE prosthesis. In addition, in vivo studies confirmed the tightness, hemocompatibility, and mechanical stability of the adhesive connection with the Dacron prosthesis. **Conclusions:** The adhesive connection between the outflow cannula and a Dacron prosthesis demonstrated superior mechanical and biological performance, including resistance to thrombogenesis and hemolysis, as well as stable integration under in vivo conditions. This solution shows high potential for safe application in the Religa Heart PED^®^ system.

## 1. Introduction

Heart failure is a clinical syndrome resulting from structural and/or functional impairment of ventricular filling or ejection, leading to perfusion inadequate to systemic metabolic demands. The condition impairs ventricular contraction and relaxation, leading to reduced cardiac output and fluid retention. As cardiac function deteriorates, the heart fails to supply adequate oxygen and nutrients to peripheral organs, ultimately compromising their function [1,2,3].

Heart failure remains a major cause of morbidity and mortality in pediatric patients and is frequently associated with congenital heart defects and cardiomyopathies [4]. The etiology in children differs from adults, with common causes including congenital heart defects such as atrioventricular septal defect, aortopulmonary window, and ventricular septal defect, the latter being the most common defect necessitating surgical intervention [5,6]. Neonatal heart failure may result from supraventricular tachycardia, severe bradycardia due to complete heart block, tricuspid valve regurgitation, or myocarditis. Additionally, metabolic disturbances like hypoglycemia, hypocalcemia, and sepsis can cause early-onset heart failure [7,8]. The working definition of pediatric heart failure characterizes it as a progressive clinical and pathophysiological syndrome resulting from cardiovascular and extracardiac abnormalities. It is marked by symptoms such as edema, respiratory distress, growth impairment, exercise intolerance, and neurohormonal dysregulation [7,8]. Treatment approaches include lifestyle interventions, pharmacological therapy, surgical repair, mechanical circulatory support (MCS), and heart transplantation [9].

Advances in pharmacotherapy and technologies used to develop MCS devices have significantly reduced mortality rates [10,11,12]. These devices, especially those serving as a bridge to transplantation or recovery, are vital to maintaining hemodynamic stability [13]. However, MCS use in pediatric patients is complex, requiring advanced technology and specialized medical teams. Most MCS devices are designed for adults and are unsuitable for pediatric use due to size constraints [14,15]. Pediatric patients present unique challenges due to continuous growth and development, often necessitating device replacement or size adjustment during long-term support. The limited thoracic space in pediatric patients precludes the use of intracorporeal rotary blood pumps, which are employed regularly in adult patients. In contrast, extracorporeal pulsatile pumps offer the advantage of being replaced without invasive surgical procedures, allowing for modular adaptation to the patient’s growth and rendering them more suitable for pediatric MCS, particularly in long-term applications [16].

The Religa Heart PED^®^ (RH PED^®^) system, developed at the Zbigniew Religa Foundation of Cardiac Surgery Development (FRK), represents Poland’s first extracorporeal pulsatile system dedicated to pediatric use. The system comprises the RH PED^®^ pulsatile pump, cannulas, and a drive unit. Extracorporeal pulsatile pumps are widely employed in patients under 16 years old as a bridge to recovery or transplantation [17]. Due to ongoing technological advancements, extracorporeal pulsatile pumps provide invaluable support in saving the lives of children and adults with severe heart conditions.

Each ventricular assist device (VAD) incorporates inflow and outflow cannulas that establish connections between the extracorporeal pump and the patient’s circulatory system, with cannula designs varying to accommodate patient age, body size, and anatomical conditions. The inflow cannula typically includes an external basket-like reinforcement that prevents suction-related collapse and maintains a stable lumen for blood flow. The outflow cannula integrates a vascular graft (commonly made of Dacron or polytetrafluoroethylene [PTFE]) that enables surgical anastomosis to the aorta and minimizes the risk of kinking or torsion during use [18]. In the pediatric population, cannulas must also allow for compatibility with smaller anatomical dimensions and remain functionally integrated over extended durations. Since the extracorporeal pump can be exchanged during therapy, the cannulas become the only permanent components in the patient’s body throughout the support period, underscoring the critical importance of their long-term stability and hemocompatibility. High flow rates of up to 5 L/min and associated shear stresses within these systems can activate coagulation pathways, increasing the risk of thrombotic complications. Ensuring stable integration of the vascular graft with the polymer cannula drain is therefore critical.

One of the primary engineering challenges is the mechanical durability of the graft-connector junction, which must withstand pressure-induced deformation and cyclic mechanical loading. In particular, Dacron grafts are prone to uncontrolled stretching and loss of structural integrity, while PTFE grafts exhibit limited compliance, elevating the risk of leakage when exposed to blood pressure fluctuations [19,20]. Additionally, since the heart chamber can be exchanged during extracorporeal support, the inflow and outflow cannulas remain the only permanent system components within the patient’s body throughout the treatment period. This necessitates exceptional long-term mechanical integrity, biocompatibility, and hemocompatibility of the connection between the polymer drain and vascular graft to ensure safe and effectual support.

This study evaluated alternative connection techniques between the polymer drain and vascular grafts for the RH PED^®^ outflow cannula. The objective was to identify a design that ensures mechanical integrity, biocompatibility, and resistance to thrombogenesis under in vitro and in vivo conditions.

## 2. Materials and Methods

### 2.1. Material Development

**Figure 1:**
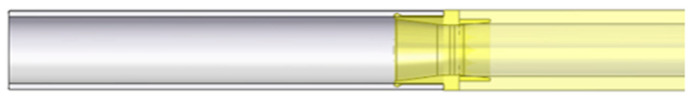
Computer-aided design schematic of the adhesive connection between the drain and prosthesis [self-developed data].

Three connection technologies for attaching vascular prostheses to the outflow cannula drain of the RH PED^®^ pediatric heart support pump were proposed for mechanical and hemocompatibility evaluation. Due to the stringent requirements of preclinical testing, all components, including pumps and connectors, were manufactured under clean-room conditions. The tested configurations included:

- adhesive connection of the drain with a Dacron (Vascutek Terumo, Inchinnan, Renfrewshire, UK) prosthesis (Figure 1);
- adhesive connection of the drain with a PTFE (GORE, Flagstaff, AZ, USA) prosthesis;
- mechanical connection of the drain with a PTFE (GORE) prosthesis.

Polymer connectors were used for adhesive configurations, while mechanical connections used metal connectors. The adhesive bonding employed an internal protocol developed by the Institute of Heart Prostheses. Each prosthesis had a diameter of 12 mm, corresponding to the RH PED^®^ pump’s outlet port and compatible with its ejection volume (stroke volume) of 45 mL. Sample sizes for each group were determined based on feasibility and alignment with commonly used in vitro and preclinical testing standards. A separate control group was not included as the study was designed to directly compare the mechanical and hemocompatibility performance of multiple clinically relevant connection types.

### 2.2. Methodology

#### 2.2.1. Tensile Strength Tests

Uniaxial tensile strength tests were performed using an MTS Criterion 45 universal testing machine to evaluate the mechanical integrity of the connections. Grips with geometry matching the connector ends were used to ensure repeatable fixation. Insert components were employed at the extremities of the samples to maintain grip rigidity. Testing proceeded until mechanical failure.

#### 2.2.2. Degradation Assessment

Durability was evaluated by exposing samples to dynamic flow conditions using simulated body fluids for a duration of t = 90 days. Test samples were arranged in a series within a dedicated test circuit equipped with a flow meter and pressure sensor. Flow was maintained at Q = 3 L/min, pressure was set at *p* = 100 mmHg, and temperature was held constant at T = 37 °C. Ringer’s solution (147.2 mmol/L Na^+^, 4 mmol/L K^+^, 2.25 mmol/L Ca^2+^, and 155.7 mmol/L Cl^−^) was used as the test medium.

#### 2.2.3. Permeability Testing

Permeability under static pressure (100 mmHg) was assessed according to PN EN ISO 7198 standards [21]. Each sample was sealed at one end and connected to the cannula drain. Measurements were taken over one-minute intervals and repeated three times per sample. Surface area (A) was calculated using Equation (1):

(1) $$A=\left(2\times \pi \times r\right)\times h$$

where r is the radius of the sample, and h is the height (length) of the prosthesis.

The calculated area was used to normalize the permeability rate, expressed in units of cm^3^/(cm^2^·min). Permeability tests were conducted using a 0.9% sodium chloride solution (154 mmol/L Na^+^ and 154 mmol/L Cl^−^) and whole human blood to mimic body fluid conditions. Each test was performed under static conditions, with *p* = 100 mmHg at T = 37 °C. The values obtained allowed for a comparative analysis of fluid leakage based on the graft type and connection method. Whole human blood was also used in parallel tests to assess permeability under physiological conditions.

#### 2.2.4. Acute Thrombogenicity Assessment

A preliminary in vitro evaluation was conducted to assess the thrombogenic potential of each connection type. Dedicated closed-loop circulatory systems were constructed for each configuration, incorporating flexible tubing, a centrifugal pump, and sensors to simulate physiological hemodynamic conditions, including flow rate (Q = 3.5 L/min), pressure (*p* = 100 mmHg), and temperature (T = 37 °C).

Porcine blood was selected for this study in accordance with a protocol established by H. Shima [22], who used porcine and human blood to evaluate acute thrombogenicity in cardiovascular devices. The use of porcine blood was further supported by the laboratory’s extensive experience with this model and the availability of validated procedures specifically adapted for porcine samples. The approach ensured consistency with previous studies and reliable interpretation of thrombogenic responses under dynamic flow conditions. Blood was collected immediately post-slaughter from healthy donor pigs at an abattoir and anticoagulated with unfractionated heparin (25 IU/mL).

The blood-filled system was monitored over time, with activated clotting time (ACT) measured using BlacT ACT Test Tubes (Hemonart Medikal Sistemler Sanayi Ticaret A.Ş., Ankara, Turkey) and a Hemonart CompactOne 9 analyzer (Hemonart Medikal Sistemler Sanayi Ticaret A.Ş., Ankara, Turkey). The test duration was defined by the moment at which ACT decreased to 1.5 times the baseline value, indicating the progressive neutralization of heparin and the potential onset of coagulation. Hematological parameters included red blood cell count (RBC, ×10^12^/L), white blood cell count (WBC, ×10^9^/L), platelet count (PLT, ×10^9^/L), hematocrit (HCT, %), and hemoglobin concentration (HGB, g/L). The analysis aimed to detect thrombocyte depletion, leukocyte–platelet interactions, or hemolytic changes resulting from contact with the tested surfaces. After the conclusion of the test, all connections were visually inspected for thrombus formation using both macroscopic and microscopic techniques.

#### 2.2.5. In Vivo Evaluation of the Connection Between the Cannula Drain and Dacron Graft

An in vivo evaluation was carried out as part of the RH PED^®^ program aimed at preparing the extracorporeal pulsatile system for clinical implementation in various pediatric sizes. The study protocol received approval from the Local Ethical Committee for Animal Experiments in Katowice, Poland. Experiments were conducted at the Center for Experimental Medicine of the Medical University of Silesia in Katowice. The study followed the principles of the 3Rs (Replacement, Reduction, and Refinement), and all procedures were designed to minimize animal discomfort and suffering.

The study employed a domestic pig model due to its cardiovascular anatomy, blood volume, and hemodynamic profile being closely analogous to those observed in human patients. Considering the 3Rs and animal welfare, one of the proposed solutions was selected for in vivo trials, including an adhesive connection with a Dacron graft. It was selected due to its surface smoothness, non-reactivity upon contact with blood, tensile strength, durability when sutured into tissues, and optimal biological response during tissue healing [23]. The objective of the in vivo study was to evaluate the functionality and biological integration of the adhesive connection between the outflow cannula drain and a Dacron vascular graft under real physiological conditions.

During the experiment, the adhesive-connected cannula assembly was surgically implanted into the circulatory system and linked to the external RH PED^®^ support pump. The system operated under pulsatile conditions, enabling functional testing of the connection during circulatory support. Parameters such as systemic pressure, flow rate, and temperature were continuously monitored throughout the procedure to ensure system stability.

The animal was euthanized at the end of the observation period using ethical protocols. The RH PED^®^ system was explanted, and macroscopic evaluation of the cannula-graft connection was performed to examine its integrity and any signs of thrombus formation or mechanical failure. Tissue samples from the anastomosis site were preserved in 4% phosphate-buffered formaldehyde for subsequent microscopic analysis, which focused on continuity, tissue response, and adhesive interface biocompatibility.

## 3. Results

### 3.1. Tensile Strength Tests

The results of the tensile strength tests are summarized in Table 1. 

**Figure 2:**
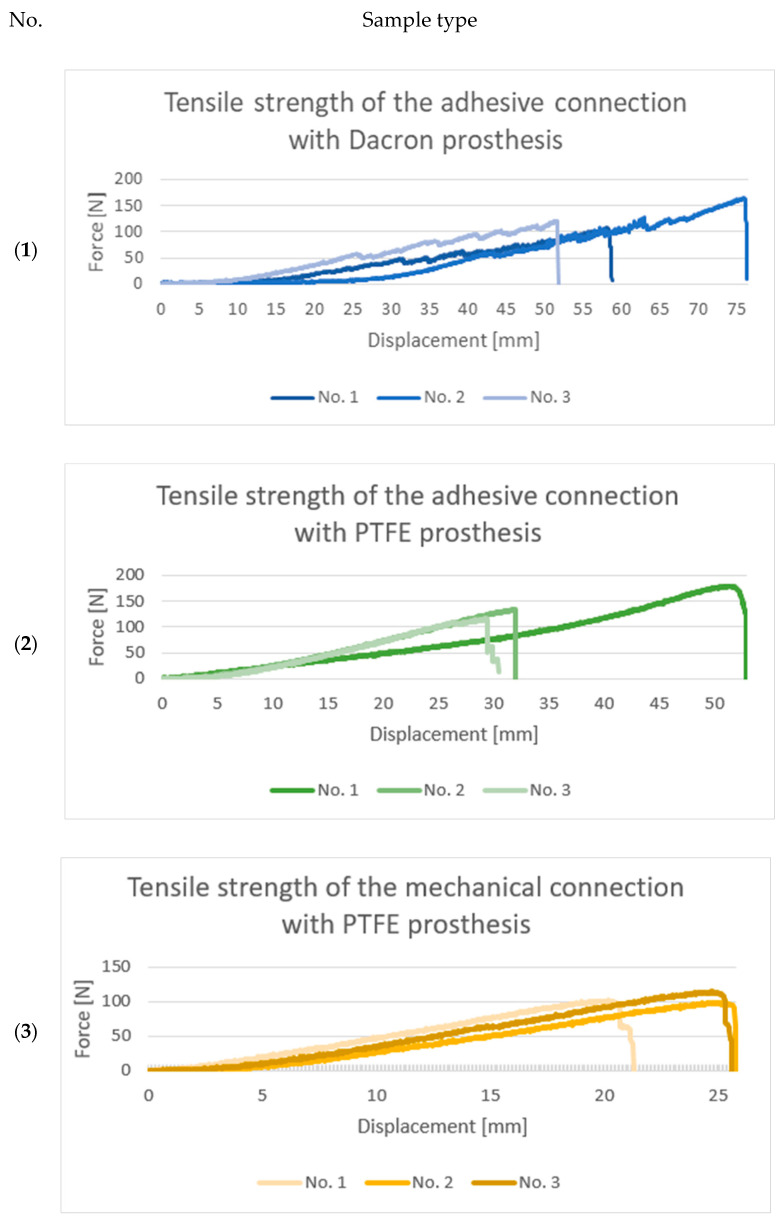
Maximum force graph recorded for the (**1**) adhesive connection with Dacron graft, (**2**) adhesive connection with polytetrafluoroethylene (PTFE) graft, and (**3**) mechanical connection with PTFE graft.

Figure 2 shows graphs comparing the recorded force values obtained from the tensile strength tests.

The adhesive connection with the PTFE graft demonstrated the highest maximum tensile strength (180 N), while the lowest value was observed for the mechanical connection with the PTFE prosthesis (99 N). The mean values followed the same trend, with the adhesive connection with the PTFE graft reaching 143 N, the adhesive connection with the Dacron graft at 131 N, and the mechanical connection with a PTFE prosthesis at 105 N. Notably, the mechanical connection with the PTFE graft samples also exhibited the lowest standard deviation (8.5 N), indicating good repeatability of this connection method.

In contrast, more variability was observed in the adhesive connections, with a 30 N standard deviation for the adhesive connection with the Dacron and 33 N for the adhesive connection with PTFE. However, this variability was not attributed to inconsistency in the connection but to prosthesis ring deformation and rupture during tensile loading. Therefore, the reported standard deviations reflect the mechanical variability of the graft materials rather than the adhesive interface.

The analysis of the force–increase curves in Figure 2. further supports this interpretation. Both PTFE-based connections—adhesive and mechanical—exhibited smooth, gradual increases in tensile force without abrupt fluctuations. In contrast, the adhesive connection with Dacron showed sudden spikes followed by sharp drops in force, indicating progressive rupture of the Dacron fiber rings. This mechanical behavior is typical of woven Dacron, where failure often initiates within the textile matrix before the adhesive interface is compromised.

**Figure 3:**
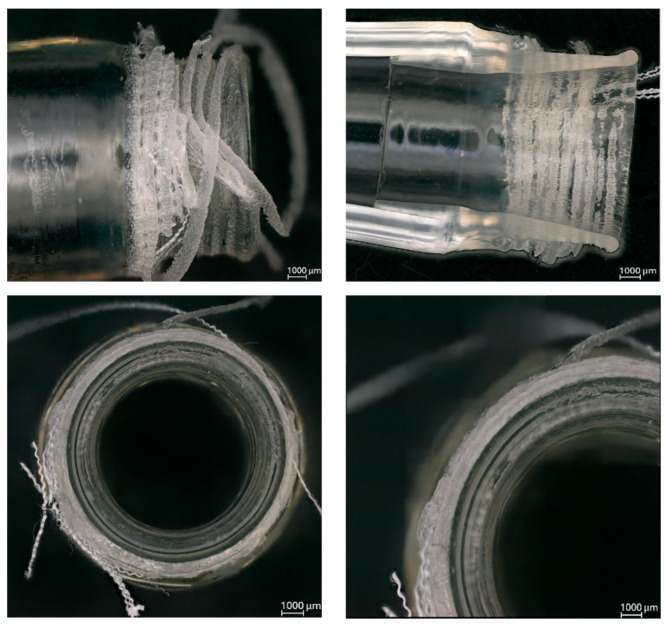
Microscopic observations after the strength test of the adhesive connection in the Dacron prosthesis, 100× magnification [self-developed data].

**Figure 4:**
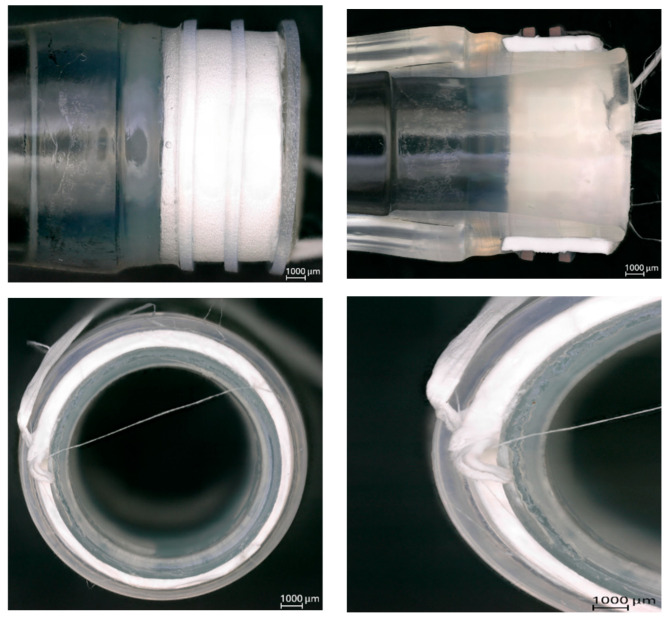
Microscopic observations after the strength test of the adhesive connection in the polytetrafluoroethylene prosthesis, 100× magnification [self-developed data].

Microscopic inspection of the adhesive samples with the Dacron graft after tensile testing (Figure 3) revealed fused Dacron fibers attached to the connector surface, confirming that material failure occurred within the graft rather than at the connection. No structural abnormalities were detected at the adhesive interface. Similar findings were observed in the adhesive connection with the PTFE graft samples (Figure 4), where the PTFE graft exhibited tearing, but the adhesive connection remained structurally intact. The interface surface was smooth and continuous, with no visible detachment between the connector, the drain, or the prosthesis.

### 3.2. Degradation Assessment

**Figure 5:**
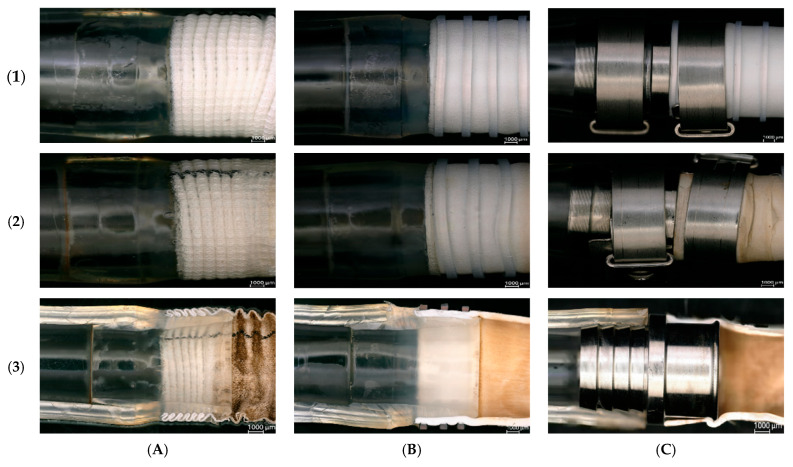
Sample degradation assessment after long-term testing. (**1**) the initial state of the samples, (**2**) the condition of samples after testing, and (**3**) an internal view of the connection. (**A**) adhesive connection with the Dacron graft, (**B**) adhesive connection with the polytetrafluoroethylene (PTFE) graft, and (**C**) mechanical connection with the PTFE graft, 100× magnification [self-developed data].

Microscopic assessment of degradation at the interface between the drain and the prosthesis was performed after 90 days of exposure to simulated physiological flow conditions. Representative images are shown in Figure 5, including the initial state of the samples (1), their condition after testing (2), and an internal view of the connection interface (3).

During the long-term test, a noticeable color change was observed in the external surface of all samples. The discoloration was directly linked to a technical malfunction of the Medtronic BPX-80 Bio-Pump (Medtronic, Minneapolis, MN, USA) which occurred on day 88 of the test. The malfunction altered the color of the circulating fluid but did not result in changes in pressure, flow, or temperature conditions, nor did it affect the structural outcome or mechanical performance of the tested connections. No evidence was found suggesting that this external artifact influenced the integrity of any evaluated parameter.

Microscopic analysis (Figure 5) did not reveal any morphological deterioration or loss of interface integrity across all tested connection types. There were no signs of delamination, adhesive separation, microcracking, or discontinuity at the junction between the polymer connector and the vascular prosthesis. The adhesive and mechanical connections remained visually intact and continuous. No debris accumulation, material erosion, or interfacial voids were detected, indicating preservation of the structural architecture throughout the testing duration.

A limited degree of fluid ingress was observed exclusively in the adhesive connection with the Dacron prosthesis sample. The accident was detected after 14 days of testing and involved minor fluid penetration through the wall of the graft. It is important to emphasize that this observation is consistent with the inherent microporous nature of woven polyester (Dacron) grafts and is recognized as a standard feature of the material’s early-phase behavior under hemodynamic loading. The phenomenon is also acknowledged by the manufacturer and does not represent adhesive failure or structural compromise at the connection interface.

No rupture events, material delamination, or performance-related degradation occurred in any of the evaluated samples throughout the 90-day test period. All tested connection types maintained mechanical stability and remained free from any macroscopic or microscopic evidence of seal loss, graft detachment, or connection discontinuity. The results confirm that both adhesive and mechanical interfaces, when subjected to long-term physiological flow simulation, exhibit robust performance characteristics with respect to structural durability and sealing function.

### 3.3. Permeability Testing

Macroscopic observations conducted during the experimental protocol revealed minor fluid permeation—approximately 1 cm^3^—through the wall of the Dacron prosthesis in the adhesive connection sample. Both saline and blood were observed to pass through the graft structure. This behavior is consistent with the known properties of woven Dacron materials and is not indicative of connection failure. Under physiological conditions, the inner surface of Dacron grafts becomes sealed by the formation of a biological lining composed of plasma proteins and early thrombus material. Until this sealing occurs, limited permeability, often referred to as “weeping,” is expected and clinically accepted.

Surface area measurements of each prosthesis were used to normalize the permeability results. The calculated surface area ranged from 16.210 cm^2^ for the adhesive connection with the PTFE prosthesis to 16.964 cm^2^ for the adhesive connection with the Dacron prosthesis (Table 2).

The permeability assessment revealed that fluid passage occurred exclusively in the adhesive connection with the Dacron prosthesis. The average permeability was calculated as 0.0589 $\frac{cm^{3}}{cm^{2}/min}$, (58.9 $\frac{mm^{3}}{cm^{2}/min}$), a low and clinically acceptable value. No measurable permeation of either saline or blood was detected in the adhesive connection with the PTFE prosthesis or in the mechanical connection with the PTFE prosthesis. These results confirm the non-porous nature of PTFE in this application and demonstrate that the tested PTFE grafts provide complete fluid retention under physiological pressure conditions.

The presence of minor permeability in the Dacron configuration does not constitute a risk under clinical use. In surgical practice, grafts of this type typically undergo intraoperative pre-clotting or are rapidly sealed post-implantation by endogenous processes. The absence of leakage at the adhesive interface, combined with the expected behavior of the Dacron material, supports the conclusion that all connection types remained sealed and functionally stable under the conditions of this test.

### 3.4. Acute Thrombogenicity Assessment

A comparative analysis of the measurements obtained during the assessment of the thrombogenic properties of the connections is presented in Table 3, Table 4 and Table 5.

Acute thrombogenicity assessment of the adhesive connection with the Dacron graft lasted for 135 min. Due to the high initial ACT value in the system, which measured 850 s, a decision was made to add 5 mg of 1% protamine sulfate at the 15 min mark of the experiment. Protamine sulfate is used to neutralize heparin, resulting in a decrease in ACT values. Following the administration of the appropriate dose, the ACT stabilized at 208 s. Subsequent measurements indicated a gradual decline in ACT values due to the degradation of heparin. An additional 1 mg of 1% protamine sulfate was added at the 90 min mark, causing a reduction in ACT to 138 s. Analysis of the blood morphological elements revealed a decrease in PLT, which may also indicate the onset of the coagulation process. The initial value of 159 × 10^9^/L decreased to 136 × 10^9^/L over the 135 min of the study.

The duration of the study on the adhesive connection with the PTFE graft was 160 min. The initial ACT of 277 s decreased to 129 s by the 160th minute of the experiment. During the study, a reduction in PLT was observed, dropping from 253 × 10^9^/L to 207 × 10^9^/L. The decrease in WBC may correlate with the decline in platelets, which could indicate the formation of leukocyte–platelet aggregates.

The study duration for the mechanical connection with a PTFE graft was 60 min. This experiment was the shortest due to a rapid drop in ACT values from 171 s to 105 s. At such a low value, the experiment had to be halted due to reaching the minimal allowable ACT threshold. Analysis of blood morphological elements showed a sudden decrease in PLT from 264 × 10^9^/L to 127 × 10^9^/L.

**Figure 6:**
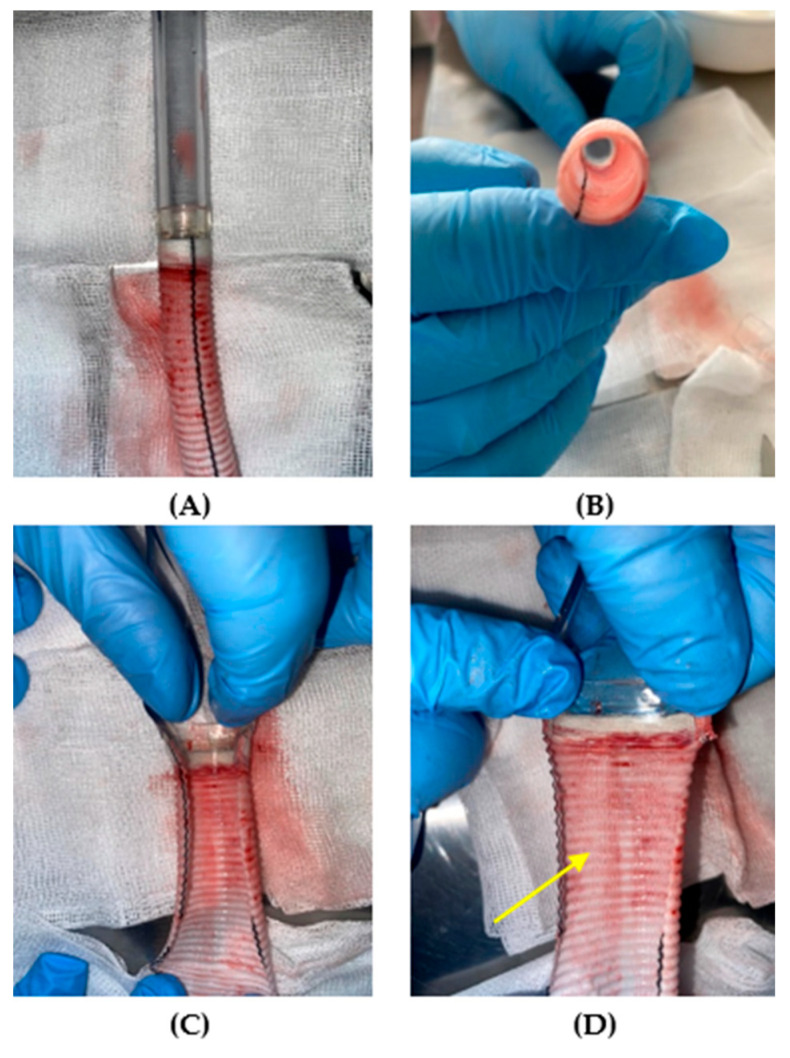
Qualitative assessment of thrombogenicity for the adhesive connection with a Dacron graft, (**A**) external view, (**B**) observation of the cannula lumen, (**C**,**D**) interior of the connection. The yellow arrow highlights the absence of thrombus between the rings of the graft [self-developed data].

**Figure 7:**
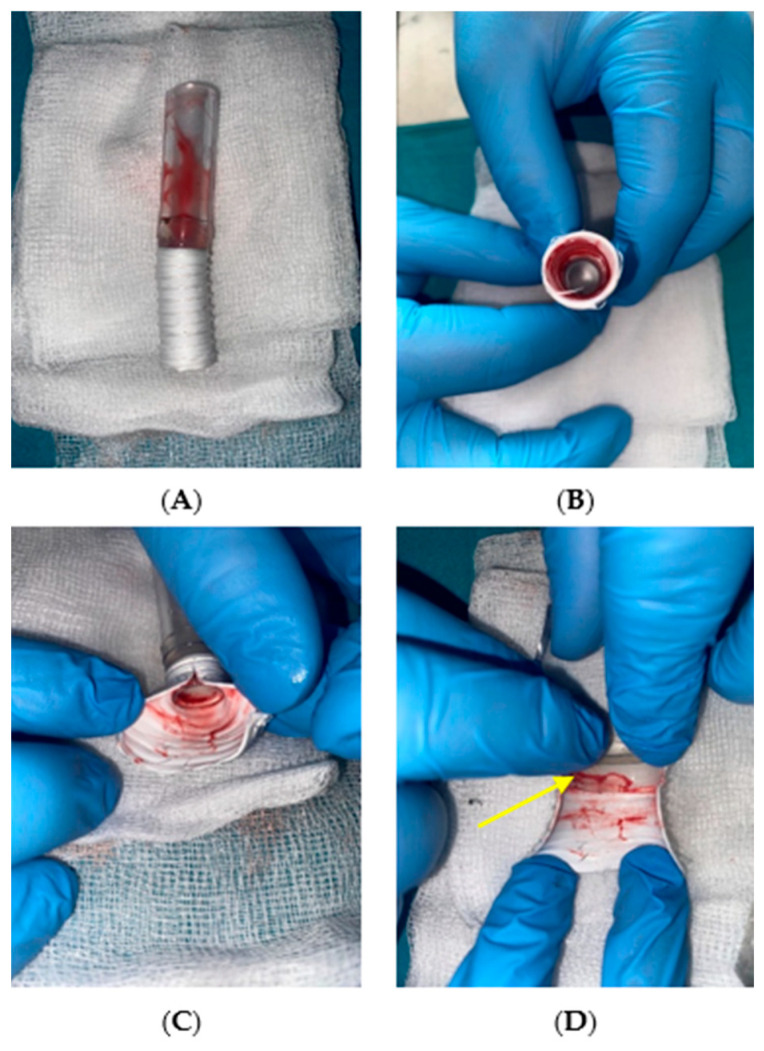
Qualitative assessment of thrombogenicity for the adhesive connection with a polytetrafluoroethylene (PTFE) graft, (**A**) external view, (**B**) observation of the cannula lumen, (**C**,**D**) interior of the connection. The yellow arrow highlights the absence of thrombus between the rings of the graft (self-developed data).

**Figure 8:**
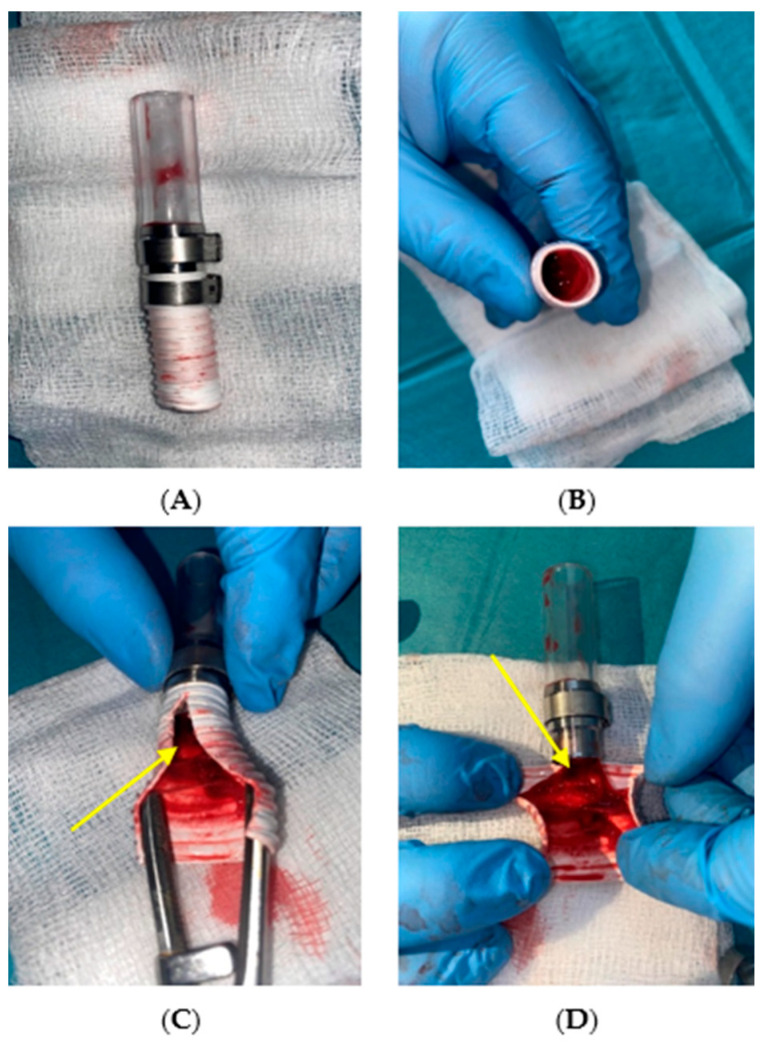
Qualitative assessment of thrombogenicity for the mechanical connection with a polytetrafluoroethylene (PTFE) graft, (**A**) external view, (**B**) observation of the cannula lumen, (**C**,**D**) interior of the connection. The yellow arrow highlights areas of thrombus formation between the rings of the graft (self-developed data).

Figure 6, Figure 7 and Figure 8 present qualitative assessments of the connections based on macroscopic observations following thrombogenicity tests.

During the qualitative evaluation of the adhesive connection with a Dacron graft, no significant changes were observed that would indicate thrombogenicity. There was no fibrous tissue overgrowth or thrombus formation within the graft rings (indicated with an arrow in Figure 6D).

Results for the adhesive connection with a PTFE graft were similar to those for the Dacron graft, with no tissue overgrowth inside the sample. The cannula lumen remained patent, and no thrombi were detected at the connection site or along the graft rings, which was also highlighted in Figure 7D.

Macroscopic evaluation of the samples revealed the presence of biological material in the mechanical connection with the PTFE graft. Thrombi were found at the junction between the connector and the graft, obstructing the graft lumen. Thrombi also built up along the graft rings, which were marked with an arrow in Figure 8C,D.

### 3.5. Evaluation of the Connection Between the Cannula Drain and Dacron Graft After In Vivo Testing

**Figure 9:**
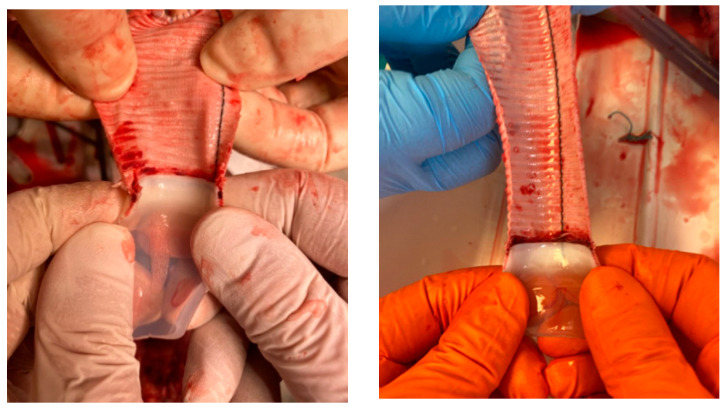
Interior of the adhesive connection of the drain with a Dacron graft outflow cannula after in vivo testing (self-developed data).

The results of macroscopic observations conducted following the explantation of the system after the observation period in the in vivo study are presented in Figure 9.

The adhesive connection between the polymer drain and the Dacron prosthesis remained sealed and mechanically stable throughout the support period. No leakage was observed at the interface, and the continuity of the inner lumen was fully preserved. Macroscopic inspection of the cannula interior revealed a smooth surface with no thrombus formation or fibrous tissue overgrowth at the connection site. The absence of intraluminal deposits indicated that the adhesive interface did not initiate coagulation processes or thromboembolic events.

**Figure 10:**
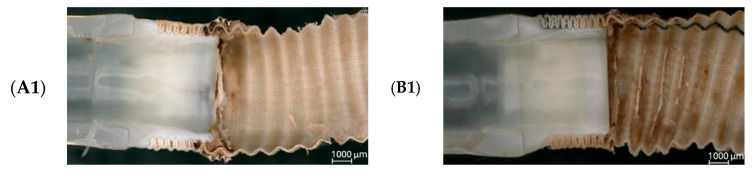

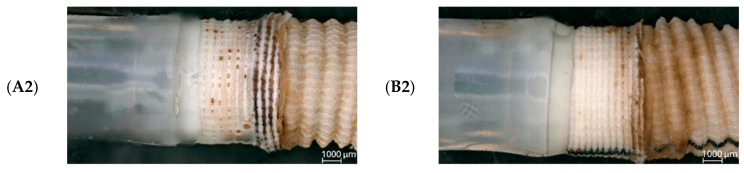
Microscopic observations of samples after in vivo testing, 100× magnification. (**A1**,**A2**) internal and external views of sample A; (**B1**,**B2**) internal and external views of sample B. (Self-developed data).

Following explantation, tissue samples from the graft–connector interface were preserved in a 4% phosphate-buffered formaldehyde solution to stabilize the biological material and prevent post-mortem degradation. These samples were subsequently subjected to microscopic examination. Figure 10 presents representative images of both the internal (1) and external (2) surfaces of the connection.

Microscopic evaluation did not reveal any structural abnormalities suggesting instability of the adhesive connection. The interfaces between the connector, drain, and prosthesis remained intact, and no delamination or detachment was detected. The adhesive connection maintained mechanical continuity without the presence of gaps or discontinuities. The internal lumen surface was smooth and regular, without abrupt transitions or geometric irregularities that could disturb blood flow or promote hemolysis.

Importantly, no tissue build-up was observed along the graft rings, either macroscopically or microscopically. This further supports the biocompatibility of the adhesive connection. There were no signs of thrombotic residue at the connection site. The geometry of the connection between the polymer components and the textile graft remained consistent, and the surface morphology showed no features suggestive of degradation or flow-induced damage.

Overall, the in vivo study confirmed that the adhesive connection with the Dacron prosthesis maintained full sealing capacity, mechanical integrity, and hemocompatibility under real physiological conditions. The connection withstood pulsatile loading without structural compromise or biological adverse reactions. These findings indicate that the adhesive interface is suitable for long-term use in the RH PED^®^ system and may offer a reliable option for clinical application in pediatric MCS.

## 4. Discussion

Macroscopic and microscopic examination of all samples prior to testing revealed no structural irregularities or manufacturing defects that could compromise the quality or performance of the evaluated connections. The adhesive connection with the Dacron prosthesis exhibited uniform bonding with no visible surface discontinuities, voids, or air inclusions. Similar observations were made for the adhesive connection with the PTFE prosthesis, which showed a consistent, smooth adhesive interface. Minor deformation at the connector notch was noted in the upper left and lower corners of some samples, attributed to compression from the polymer connector geometry, but this had no impact on functional performance.

The results of tensile strength tests, shown in Table 1, indicated that the highest breaking force was observed in the adhesive connection with the PTFE graft (180 N), while the lowest force was recorded for the mechanical connection sample (99 N). Average breaking forces were similar, with an average of 143 N for the adhesive connection with the PTFE prosthesis and 105 N for the mechanical connection with the PTFE prosthesis. Strength testing confirmed that, for adhesive connections with both Dacron and PTFE prostheses, the graft itself ruptured rather than the adhesive connection site, indicating that the connection strength exceeds the strength of the materials used in vascular prostheses. The maximum breaking forces (165 N for the adhesive connection with the Dacron prosthesis and 180 N for the adhesive connection with the PTFE prosthesis) thus reflect graft strength rather than connection strength.

A drawback of the adhesive connection with the PTFE prosthesis is the susceptibility of the PTFE graft to mechanical damage, such as during clamping in the testing machine. The mechanical connection samples with the PTFE prosthesis proved to be the least resistant to tensile forces. The advantage of this type of connection lies in its simplicity and rapid preparation, which is significant for optimizing the manufacturing processes of MCS components. The test results indicate sufficient values—each proposed connection can withstand the forces present within the circulatory system and is resistant to potential medical handling errors (e.g., accidental drain pulling). Generating a force equal to 99 N, the minimum breaking force recorded during testing, is highly unlikely during surgery or spontaneous movements.

Microscopic observations of samples after tensile testing further supported these conclusions. In both adhesive configurations, it was the prosthesis rather than the connection that failed under load. In the adhesive connection with the Dacron prosthesis (Figure 3), ruptured fibers were clearly visible, while the adhesive interface remained unaffected. Similarly, in the adhesive connection with the PTFE prosthesis (Figure 4), the graft material showed structural damage, but the adhesive connection maintained continuity. No detachment between the connector and the drain or prosthesis was observed in any of the tested samples.

The assessment of long-term degradation under simulated physiological flow conditions showed no structural damage or material deterioration in any of the tested connection types over the 90-day exposure period (Figure 5). A change in fluid coloration was observed after day 88 due to a malfunction of the Medtronic BPX-80 Bio-Pump. However, this event had no measurable impact on mechanical integrity, sealing performance, or sample morphology. The macroscopic and microscopic evaluation confirmed that the interfaces in all configurations (adhesive and mechanical) remained continuous and undisturbed. No rupture, detachment, or leakage was detected at any point during or after testing. In the sample with the adhesive connection with the Dacron prosthesis, slight permeation of the test solution through the graft wall was noted beginning on day 14. This is a known behavior of woven polyester materials and is explicitly stated in the manufacturer’s specifications. Importantly, this fluid transfer did not originate at the adhesive interface and did not affect the seal of the connection itself.

During permeability testing, only the sample with the adhesive connection to the Dacron prosthesis exhibited slight seepage of saline and blood through the graft (1 cm^3^/min). However, this is a natural phenomenon during implantation in which a small amount of blood also seeps through the graft until the prosthesis is sealed by blood protein deposition. After approximately 14 days, the gelatin sealant in the graft undergoes hydrolysis and is replaced by tissue overgrowth. The permeability of the sample with the adhesive connection to the Dacron prosthesis was 0.0589 $\frac{cm^{3}}{cm^{2}/min}$, indicating very low permeability (Table 2). For both types of samples with the PTFE prosthesis, neither saline nor blood permeated the prosthesis material.

The purpose of the acute thrombogenicity study was to analyze the thrombogenic properties of the connections under in vitro flow conditions simulating the target application site. Porcine blood obtained during slaughter was used as the testing medium. The experiment duration for the adhesive connection with the Dacron prosthesis was 135 min (Table 3). Qualitative analysis after the study showed that the adhesive connection sample with the Dacron prosthesis was free of thrombi, and no fibrous tissue build-up or graft ring filling with clots was observed (Figure 6). The study also allowed for assessing the connection’s permeability under dynamic flow conditions. Initially, the Dacron graft exhibited “sweating,” a natural phenomenon for Dacron grafts. After approximately 15 min, the graft was sealed internally by blood, halting seepage. For the adhesive connection sample with the PTFE prosthesis, after initiating circulation in the setup, a gradual decrease in ACT was observed due to heparin breakdown, from 277 s to 129 s at 160 min (Table 4). Macroscopic evaluation of the adhesive connection sample with the PTFE prosthesis revealed no thrombi at the connection site or along the graft rings (Figure 7). No blood seepage through the PTFE graft material was observed in either the adhesive or mechanical connection samples. In the acute thrombogenicity study of the mechanical connection with the PTFE prosthesis, the experiment lasted 60 min due to a rapid decline in ACT values from 171 s to 105 s within 60 min (Table 5). Qualitative analysis of the mechanical connection sample with the PTFE prosthesis after the experiment revealed the presence of biological material. Thrombi were observed at the connector–prosthesis junction, obstructing the prosthesis lumen (Figure 8). Observations of the thrombus location indicated that the process of thrombus formation was not influenced by leading edges between the connector and drain, confirmed by the absence of thrombi in these areas.

The results of the in vivo evaluation provided additional confirmation of the mechanical and biological performance of the adhesive connection with the Dacron prosthesis. Throughout the duration of pulsatile support in the porcine model, the connection remained sealed and structurally stable. No leakage, mechanical disruption, or thrombus formation was observed at the interface between the polymer drain and the vascular graft. Macroscopic examination after system explantation revealed a continuous, smooth internal surface with no fibrous tissue accumulation or signs of flow-induced damage (Figure 9).

Microscopic analysis of samples preserved in phosphate-buffered formaldehyde further confirmed these findings. The adhesive interface between the connector, drain, and prosthesis was morphologically intact, with no evidence of delamination, discontinuity, or biological overgrowth (Figure 10). The internal lumen of the connection remained free of surface irregularities or protrusions that could promote hemolysis or thrombogenesis.

The preserved integrity and smooth internal profile of the adhesive interfaces observed in this study are consistent with broader findings in the literature regarding the role of connection geometry in thrombogenic risk. Mendez Rojano et al. demonstrated through computational modeling that low velocity and disturbed shear flow conditions at cannula interfaces can initiate thrombus formation, even in anatomically distinct inlet configurations [24]. Similarly, Neidlin et al. reported that variations in cannula alignment affected intraventricular flow patterns, residence time, and washout efficiency, all of which are relevant to thrombus development [25]. Although these studies focused on inflow scenarios, their conclusions reinforce the general importance of interface geometry and flow continuity, principles that are clearly reflected in the thromboresistant behavior of the adhesive connections in the present study.

This study has certain limitations. The in vivo tests were performed in a porcine model, which has coagulation and hemodynamic properties that enable clinically relevant evaluation of cardiovascular interfaces. However, interspecies differences limit direct translation to pediatric patients. In addition, the test duration was limited to 30 days, which does not allow assessment of long-term integration. Further in vivo studies of extended duration, followed by clinical evaluation, are needed to validate this connection method fully. All samples were sterilized prior to testing, as required for internal biomedical use. Although the potential influence of sterilization on material properties was not evaluated, the procedure reflects standard preparation for implantable components.

Based on these results, the adhesive connection between the outflow cannula drain and a Dacron prosthesis demonstrated the most favorable combination of mechanical strength, sealing integrity, and hemocompatibility. The findings support its potential suitability for integration into the RH PED^®^ system and justify further investigation in extended in vivo studies and prospective clinical applications.

## 5. Conclusions

Based on the research conducted, all proposed connections were characterized by sufficient tensile strength in terms of use. The adhesive connection was characterized by the highest tensile strength with a PTFE prosthesis. The lowest tensile strength was observed for the mechanical connection with the PTFE prosthesis. Microscopic observations of the samples after strength tests did not reveal any structural changes that could indicate the instability of the connections. Microscopic observations of samples after long-term testing revealed no evidence of anastomotic degradation or loss of seal integrity. Post-test, samples remained morphologically consistent with their initial state. All proposed configurations exhibited minimal fluid permeability, and no clinically relevant leakage was observed that could compromise patient safety.

The adhesive connection with the Dacron prosthesis and the adhesive connection with the PTFE prosthesis showed no signs of thrombogenicity. The thrombus formation observed in the mechanical connection with the PTFE prosthesis may be associated with interface-related factors, such as material properties or local flow disturbances. In contrast, adhesive connections form smooth, continuous bonds that may reduce conditions associated with thrombus development. Adhesive connection of the drain with a Dacron prosthesis to the outflow cannula used during in vivo studies showed no susceptibility to thrombogenesis or hemolysis. The adhesive connection of the drain with the Dacron prosthesis to the outflow cannula used during in vivo tests ensured connection tightness without the risk of tearing or disconnecting both structures. Based on the conducted research, the solution has the potential to be safely used in the RH PED^®^ system.

These findings provide a basis for the clinical translation of adhesive connection technology in pediatric circulatory support. Future studies should focus on chronic in vivo validation and optimization for manufacturing scalability and clinical integration. Among the tested configurations, adhesive connections demonstrated the most favorable performance in vitro, and the in vivo results with the adhesive connection with the Dacron prosthesis further support the continued development of this approach for pediatric circulatory support applications. This connection method may be particularly useful in pediatric patients requiring stable and hemocompatible integration of vascular grafts, such as during surgical assembly of pulsatile support systems.

## Figures and Tables

**Table 1:** Maximum tensile force values obtained for individual samples during the tensile strength test.

	Recorded Force [N]		
Sample No.	Adhesive Connection with Dacron Prosthesis [N]	Adhesive Connection with PTFE Prosthesis [N]	Mechanical Connection with PTFE Prosthesis [N]
1	121	180	102
2	165	134	99
3	107	116	115
Average	131	143	105
Standard dev.	30	33	8.5

**Table 2:** Results of the permeability testing.

Parameter	Adhesive with Dacron Prosthesis [N]	Adhesive with PTFE Prosthesis [N]	Mechanical with PTFE Prosthesis [N]
Amount of permeated fluid [cm^3^/min]	1	0	0
$\mathrm{Permeability}\;[\frac{cm^{3}}{cm^{2}/min}]$	0.0589	0	0

**Table 3:** Comparative analysis of morphological elements and activated clotting time (ACT) for adhesive connection with a Dacron graft.

Time [min]	WBC [×10^9^/L]	RBC [×10^12^/L]	HGB [g/L]	HCT [%]	PLT [×10^9^/L]	ACT [s]
0	5.8	5.81	94	31.0	159	850 ^a^
35	6.2	5.88	99	31.2	144	208
45	6.0	6.20	100	33.2	158	202
60	6.0	5.98	99	31.9	137	207
90	5.8	6.20	100	33.0	135	216
105	5.8	6.13	99	32.7	136	138
135	5.7	6.05	100	32.5	136	114

^a^ (The threshold value for the addition of protamine sulfate).

**Table 4:** Comparative analysis of morphological elements and activated clotting time (ACT) for the adhesive connection with a polytetrafluoroethylene (PTFE) graft.

Time [min]	WBC [×10^9^/L]	RBC [×10^12^/L]	HGB [g/L]	HCT [%]	PLT [×10^9^/L]	ACT [s]
0	9.6	5.54	92	31.4	253	277
30	-	-	-	-	-	216
60	9.8	5.30	90	30.2	220	181
80	-	-	-	-	-	147
90	9.7	5.28	89	30.0	203	145
115	9.4	5.61	92	31.8	205	149
140	9.2	5.64	93	32.3	203	148
155	-	-	-	-	-	142
160	9.0	5.38	92	30.9	207	129

**Table 5:** Comparative analysis of morphological elements and activated clotting time (ACT) for the mechanical connection with a polytetrafluoroethylene (PTFE) graft.

Time [min]	WBC [×10^9^/L]	RBC [×10^12^/L]	HGB [g/L]	HCT [%]	PLT [×10^9^/L]	ACT [s]
0	14.8	6.84	111	38.3	264	171
15	-	-	-	-	-	150
30	14.5	6.60	108	37.0	127	110
60	-	-	-	-	-	105

## Data Availability

The original contributions presented in this study are included in the article. Further inquiries can be directed to the corresponding author.
